# Cleavage of the HPV16 Minor Capsid Protein L2 during Virion Morphogenesis Ablates the Requirement for Cellular Furin during *De Novo* Infection

**DOI:** 10.3390/v7112910

**Published:** 2015-11-11

**Authors:** Linda Cruz, Jennifer Biryukov, Michael J. Conway, Craig Meyers

**Affiliations:** Department of Microbiology and Immunology, The Pennsylvania State University College of Medicine, Hershey, PA 17033, USA; lcruz@hmc.psu.edu (L.C.); jhopkins@hmc.psu.edu (J.B.); conwa2m@cmich.edu (M.J.C.)

**Keywords:** human papillomavirus (HPV), HPV16, furin, J0101

## Abstract

Infections by high-risk human papillomaviruses (HPV) are the causative agents for the development of cervical cancer. As with other non-enveloped viruses, HPVs are taken up by the cell through endocytosis following primary attachment to the host cell. Through studies using recombinant pseudovirus particles (PsV), many host cellular proteins have been implicated in the process. The proprotein convertase furin has been demonstrated to cleave the minor capsid protein, L2, post-attachment to host cells and is required for infectious entry by HPV16 PsV. In contrast, using biochemical inhibition by a furin inhibitor and furin-negative cells, we show that tissue-derived HPV16 native virus (NV) initiates infection independent of cellular furin. We show that HPV16 L2 is cleaved during virion morphogenesis in differentiated tissue. In addition, HPV45 is also not dependent on cellular furin, but two other alpha papillomaviruses, HPV18 and HPV31, are dependent on the activity of cellular furin for infection.

## 1. Introduction

Papillomaviruses (PVs) encompass a large family of non-enveloped DNA viruses. Human papillomaviruses (HPVs) are medically important as infection by the high-risk HPV types are causative agents of malignancies in the cervical epithelium, other anogenital sites, as well as in the oral mucosa [1,2]. The viral capsid consists of 72 pentamers of the major capsid protein, L1, which surrounds the circular, double-stranded, 8-kb viral genome [3]. The L1 protein serves as the main structural protein as it alone can self-assemble to form viral particles [4]. L1 has also been shown to function in primary attachment to the host to initiate infection [5,6]. Potentially up to 72 copies of the minor capsid protein, L2, associate with the L1 capsomers and are thought to occupy and partially extrude through the center of the L1 pentamers [7,8]. The L2 protein serves many roles during infections [9]. L2 has a structural role as it aids in genome encapsidation and stabilization of the viral particles [10,11,12]. During entry, L2 interacts with several host proteins, resulting in receptor engagement, escape from the endosomal compartments, trafficking through the Golgi network, and finally escorting the viral genome to the nucleus [13,14,15,16,17,18,19,20,21,22,23,24]. Cleavage of L2 at a conserved furin cleavage consensus site in the N-terminus by the proprotein convertase (PC) furin or the furin-like protease PC 5/6 has been shown to be a hallmark feature during pseudovirus (PsV) entry and is required for endosomal escape and productive infection. Cleavage of L2 takes place post heparan sulfate (HS) attachment on the cell surface or the extracellular matrix (ECM) [25,26]. Many other viruses have been shown to require the activity of furin-like proteases for their infectivity. However, rather than playing a role during entry, these enveloped particles are cleaved during virion maturation while transiting through acidic compartments of the secretory pathways prior to release of the particles and attachment to uninfected host cells [27,28,29,30].

HPVs infect mucosal and cutaneous stratified squamous epithelia and the viral life cycle and new virion production is strictly dependent on host-tissue differentiation [31]. Previous research on the entry process of HPV into its host cells has largely been performed using recombinant PsV particles. PsVs are produced by expression of the viral capsid proteins, L1 and L2, in monolayer cells and do not go through the natural maturation process. In addition, they incorporate a reporter plasmid in place of the histone-associated viral genome [32]. HPV16 PsV has been utilized to elucidate many of the details of the HPV entry pathway [33]. PsV particles resemble authentic virions as they have similar structure by cryoelectron microscopy and they retain the majority of surface-exposed conformational-dependent epitopes [8,34,35,36,37]. L1 only virus-like particles elicit a strong antibody response, which is efficient against authentic virions and are the basis for the current vaccines [38]. However, it is unclear what structural differences may exist and how this may affect the biology and entry pathway of the virus [39]. The slow maturation process of native virions (NVs) in a differentiating epithelium plays an important role in regulation of capsid formation and conformational changes over time. Antibody-mediated neutralization of L1 and L2 epitopes is enhanced over a 10-day period as the particles become more mature, suggesting that controlled conformational changes occur in context of the differentiating tissue. The cellular environment and cellular factors influence the final structure of tissue-derived particles [40,41,42,43,44,45]. Cross-neutralization by an array of L2 specific antibodies targeting surface-exposed epitopes of L2 displayed a different pattern of neutralization for tissue-derived particles as compared to previously published data on PsV particles, further supporting the existence of some structural differences [46]. Temporal changes in L2 exposure on the virion surface have been revealed through neutralization of HPV16 PsV with the monoclonal antibody RG-1 (amino acids 17–36). Exposure of the L2 N-terminus along with the furin cleavage site is masked in the mature PsV particles and is only displayed hours post-attachment to host cells [25,47]. In addition, neutralization by RG-1 of tissue-derived NV particles is inefficient when immature 10-day virions are extracted as compared to the efficient neutralization observed of mature 20-day virions [40]. These results suggest that conformational changes may occur during virion morphogenesis as well as during infection.

Recently, we showed that high-risk HPV NV types display different patterns of dependence on glycosaminoglycans (GAGs) for infection [48]. This differential use of GAGs for cell surface attachment by different HPV types was then confirmed utilizing HPV quasivirus (QV). Specifically, HPV16 was shown to be able to attach to cells independent of GAGs, instead attaching to laminin-332 [49]. Of particular interest, HPV16 NV was not blocked by any GAG tested and was insensitive to the sulfation status of the GAGs [48]. In contrast, all PsVs tested to date are dependent on GAGs, specifically HS, for infection [25]. A close relationship between L1-mediated primary attachment to HS followed by cleavage of the L2 N-terminus by furin has been demonstrated for the infectious entry of HPV16 PsV [26,50]. We hypothesized that the L2 protein of GAG-independent HPV16 NV may be cleaved during maturation in tissue, thus allowing for furin-independent entry and infection by HPV16 NV. We show here that HPV16 NV is not blocked by a furin inhibitor and infects furin and PC 5/6-deficient cells as efficiently as cells expressing furin. Further, we show that raft-derived HPV16 particles may contain full-length as well as cleaved L2 protein.

## 2. Results

### 2.1. HPV16 NV Is Independent of Cellular Furin and Furin-Related Proprotein Convertases for Infection of HaCaT Keratinocytes

**Figure 1:**
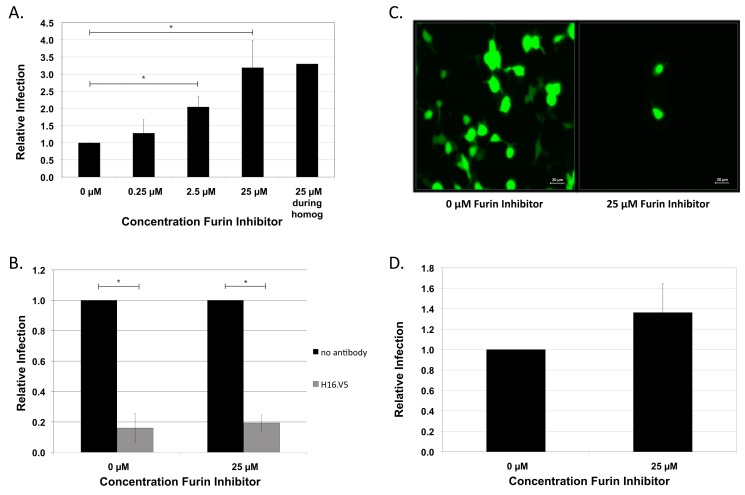
Furin-independent HPV16 NV infection. (**A**) Infection of HaCaT cells with foreskin-derived HPV16 at increasing concentrations (0.25 µM, 2.5 µM, 25 µM) of the furin peptide inhibitor. The infection at 25 µM inhibitor concentration was also repeated after harvesting the virus in the presence of 25 µM inhibitor; (**B**) Virus preparations were incubated with 1:100 dilutions of H16.V5 antibody for 1 h prior to infections in the absence or presence of 25 µM furin inhibitor; (**C**) HPV16 PsV and (**D**) HPV16 NV infections of 293TT cells in the absence or presence of 25 µM furin inhibitor. HPV16 PsV infection was assessed by immunofluorescent microscopy monitoring GFP-expression two days post-infection. HPV16 NV infections were analyzed by RT-qPCR measuring the relative amount of E1^E4 transcript two days post-infection. The data is plotted as relative infection at the different concentration with infection at 0 µM furin inhibitor set equal to one. The results are expressed as the means of at least three independent infections utilizing at least two different virus preps and standard deviations are shown. Statistical significance (denoted by asterisks) was determined by student’s *t*-test and significance was defined as *p* ≤ 0.05.

To determine whether native HPV16 produced in differentiating epithelial tissue is dependent on cellular furin, we infected HaCaT cells, a human spontaneously immortalized keratinocyte cell line, in the presence or absence of increasing amounts of a furin inhibitor. HPV16 NV produced in differentiating foreskin tissue was previously characterized [40]. HPV16 NV infection was not inhibited, but, rather, a dose-dependent increase in infectivity was observed in response to increasing concentrations of the furin inhibitor (Figure 1A). In order to obtain a pure, mature, more homogenous preparation of particles, recombinant HPV particles are generally purified via ultra centrifugation through an opti-prep gradient prior to use. To be sure that HPV16 NV particles were not insensitive to furin inhibition due to a heterogenous mixture of virions as well as proteins in the homogenate, infections were done utilizing opti-prep purified HPV16 NV in the presence of furin inhibitors. To rule out the possibility that the virus was processed by residual PC activity in the homogenate during virus harvest (homogenization and benzonase-treatment) we added the furin inhibitor during harvest. Addition of the inhibitor had no effect as compared to when no inhibitor was added during harvest (Figure 1A). To confirm previous data that infection of HaCaT keratinocytes by HPV16 is particle-mediated [40,48], and to ensure that addition of the furin inhibitor to the cells does not cause uncharacterized virus uptake, infections were done in the presence of the L1-specific, conformation-dependent, monoclonal antibody H16.V5 [51,52]. Indeed, infection in the presence of the furin inhibitor is particle-mediated supporting that *in vitro* infection by tissue-derived HPV16 is a furin-independent process (Figure 1B). As this result is in stark contrast to previous data using HPV16 PsV, we wanted to confirm previously shown data using our experimental conditions. We used 293TT cells due to the superior signal intensity by GFP-expressing PsV in this cell line [53]. Addition of the furin inhibitor during PsV infection of 293TT cells nearly completely ablated the GFP signal (Figure 1C), confirming that *in vitro* infection by HPV16 PsV is a furin-dependent process. The ability to infect cells in the absence of active cellular furin was not cell-type dependent as infection of 293TT cells by HPV16 NV was also unable to be blocked by the furin inhibitor (Figure 1D). The furin inhibitor also blocks substrate-binding to other related PCs [54] suggesting that HPV16 NV does not require proteolytic cleavage by any furin-related PC for efficient entry during infection.

### 2.2. HPV16 NV Can Infect Furin-Deficient Cells

**Figure 2:**
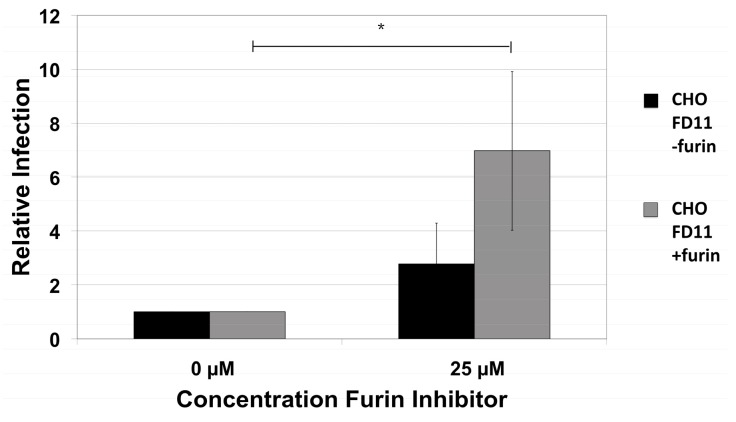
Infection of furin-negative CHO FD11 (−furin) cells and furin-positive CHO FD11 (+furin) cells. Infection of furin-negative and the furin-positive CHO FD11-derivative cell lines with HPV16 in the presence or absence of 25 µM furin inhibitor. Infections were analyzed by RT-qPCR measuring the relative amount of E1^E4 transcript two days post-infection normalizing to infection by the CHO parental cells. The results are expressed as the means of at least three independent infections utilizing at least two different virus preps and standard deviations are shown. Statistical significance (denoted by asterisks) was determined by student’s *t*-test and significance was defined as *p* ≤ 0.05.

To further characterize the furin-independence by HPV16 NV and avoid potential off-target effects by the furin inhibitor, we performed infections with furin-negative cells. CHO FD11 cells have a genetic mutation that eliminates expression of furin and they also do not express PC5/6 [55]. HPV16 NV infected CHO FD11 cells at similar levels to that of a furin-expressing derivative CHO FD11 + furin cell line [55]. Addition of the furin inhibitor during infection with HPV16 did not change infection of the furin-negative cells, but, rather, an increase in infection was observed in the furin-positive cells when the inhibitor was present (Figure 2).

### 2.3. Exogenous Furin Has No Impact on Infection

**Figure 3:**
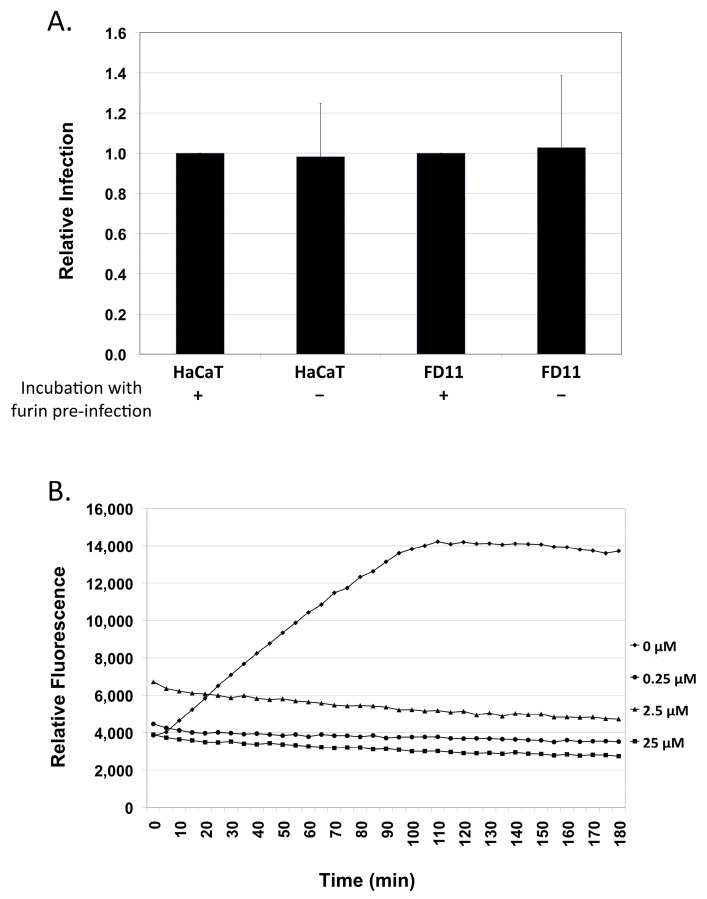
Addition of exogenous furin prior to infection has no effect on infectivity. (**A**) Infection of HaCaT cells and furin-negative CHO FD11 cells after incubating the virus for 7 h at 37 °C with 5U exogenous furin enzyme. Infections were analyzed by RT-qPCR measuring the relative amount of E1^E4 transcript two days post-infection normalizing to virus infections not treated with exogenous furin; (**B**) Cleavage of the Boc-Arg-Val-Arg-Arg-AMC fluorogenic peptide at increasing concentration (0, 0.25, 2.5 and 25 µM) of furin inhibitor in virus 0.05 M phosphate buffer. Fluorescence was recorded at Ex/Em 350/450. The results are expressed as the means of at least three independent infections utilizing at least two different virus preps and standard deviations are shown. Statistical significance (denoted by asterisks) was determined by student’s *t*-test and significance was defined as *p* ≤ 0.05.

While insensitive to the addition of a furin inhibitor at the time of and during infections, it remained possible that furin may be able to impact the virus prior to infection. We first investigated this by incubating the virus with exogenous furin prior to infection. Incubation of the virus preps with exogenous furin prior to infection of HaCaT or CHO FD11 cells failed to enhance infection (Figure 3A). This is in contrast to HPV16 PsV, where pre-treatment with exogenous furin may enhance infection of furin-deficient as well as furin-expressing cells [50]. To verify that the furin enzyme and inhibitor were fully functional in our assay conditions, an *in vitro* cleavage assay using the Boc-RVRR-AMC fluorogenic peptide containing a furin cleavage site was performed. The assay was done in the same buffer conditions as the viral preparations as well as in the presence of cellular lysate from HPV negative foreskin rafts. The fluorogenic peptide was efficiently cleaved by exogenous furin and was effectively blocked by the inhibitor even at the lowest concentration, suggesting the assay conditions are not responsible for the observed results (Figure 3B).

### 2.4. Expression of Furin in Organotypic Foreskin and Cervical Cultures

**Figure 4:**
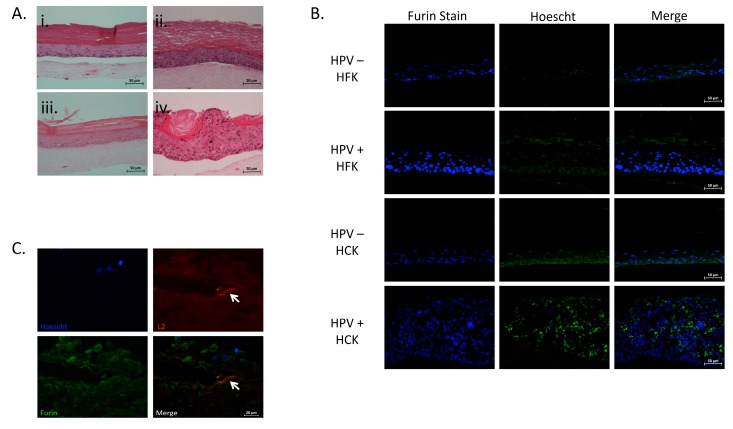
Expression of furin in raft cultures. Hematoxylin and eosin (H&E) staining (**A**) of HPV-negative primary foreskin tissue (i), HPV16-positive foreskin tissue (ii), HPV-negative primary cervical tissue (iii), and HPV16-positive cervical tissue (iv); Immunofluorescent staining (**B**) using the furin (MON-148) antibody of HPV-negative primary foreskin tissue (**top row**), HPV16-positive foreskin tissue (**top-middle row**), HPV-negative primary cervical tissue (**bottom-middle row**), and HPV16-positive cervical tissue (**bottom row**). Nuclear stain was done using Hoechst 33342. Furin stain (**green**). Nuclear stain (**blue**). HPV16-positive foreskin tissue co-stained with furin (MON 148) and L2 (RG-1) antibody (**C**). Nuclear stain was done using Hoechst 33342. Furin stain (**green**). L2 stain (**red**). Nuclear stain (**blue**). White arrows indicate L2 positive cells. Sections were harvested from tissues grown for 20 days in organotypic raft cultures. All images are representative of two sets of staining in two individual sets of rafts.

Due to the strict differentiation-dependent tissue tropism of PVs, we were interested in the furin expression in virus-infected organotypic epithelium. Expression of furin throughout the epithelium has been shown in sections of human epithelium [56]. Expression and localization of furin in the intact mouse genital tract indicates the presence of furin through all layers of the epithelium. Following wounding, the furin expression is intensified, particularly in the basal layer [26]. Organotypic cultures may resemble to some extent a wound-healing environment as the cultures are initiated as a single layer before proliferation, stratification, and differentiation takes place. Primary human foreskin (HFK) and cervical (HCK) keratinocytes are both physiologically relevant cells for the maintenance, spread, and pathogenesis of HPV. To determine the effect of virus infection and replication in foreskin and cervical tissue, we grew HPV-negative as well as HPV16-positive rafts from both tissues (Figure 4Ai, ii, iii and iv). The expression of furin in HPV-negative foreskin- and cervix-derived organotypic raft cultures was diffuse throughout the basal and lower nucleated suprabasal layers, with more intense staining observed in the basal layer (Figure 4B). In HPV16-positive foreskin- and cervix-derived tissues, there was an overall higher intensity of furin with staining observed primarily in the upper layers of the epithelium. A small amount of furin was present in the basal cells (Figure 4B). Furin was also shown to co-localize with L2 in HPV-positive foreskin-derived tissue (Figure 4C).

**Figure 5:**
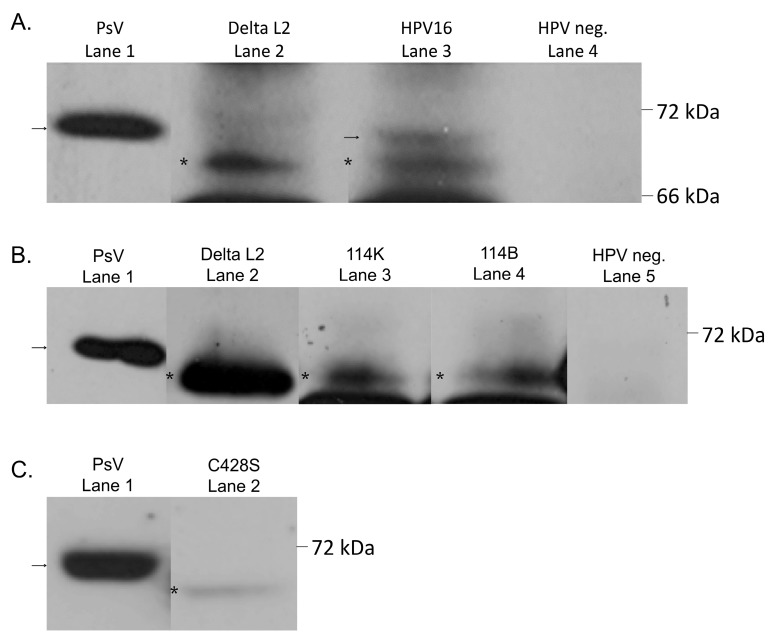
Cleavage of the L2 capsid protein from native virus. (**A**) Western blot analysis of HPV16 L2. Lane 1: HPV16 PsV, lane 2: HPV16 L2 expression plasmid with N-terminal deletion, lane 3: HPV16 114B NV, lane 4: HPV-negative primary raft lysate; (**B**) Western blot analysis of HPV16 L2 from two European isolates. Lane 1: HPV16 PsV, lane2: HPV16 L2 expression plasmid with N-terminal deletion, lane 3: HPV16 114K NV, lane 4: HPV16 114B NV, lane 5: HPV-negative primary raft lysate; (**C**) Western blot analysis of an HPV16 L1 mutant virus. Lane 1: HPV16 PsV, lane 2: HPV16 C428S NV. The arrows and asterisks indicate the two forms of L2 species observed in tissue-derived NV particles.

### 2.5. Cleavage of HPV16 during Virion Morphogenesis

Since furin and PC 5/6 are abundantly expressed in the keratinocyte epithelium, the natural target for HPV infection, we were interested if the native virus might be processed by furin during assembly, before cell attachment and entry. Expression of the L1 and L2 capsid proteins occur only in the suprabasal layers and has been previously shown for wild-type HPV16 organotypic cultures [40,57]. Because a higher level of furin expression in the suprabasal layers coincides with the production of HPV capsid proteins, we hypothesized that the HPV16 L2 minor capsid protein may be cleaved during virion maturation. To examine the state of the L2 protein in tissue-derived HPV16 virus particles, we probed the harvested virus preparations separated on an SDS-PAGE gel with the RG-1 L2 monoclonal antibody. HPV16 PsV L2 migrated at about 70 kDa as previously shown, despite its calculated molecular size of about 55 kDa (Figure 5, lane 1) [58,59,60,61]. A faster-migrating L2 species was observed in HPV16 NV viral preparations derived from organotypic raft cultures (Figure 5A, lane 3). The size of the faster-migrating L2 species was verified by an N-terminally modified L2 expression-plasmid lacking amino acids 2–12, corresponding to the consensus furin cleavage site (Figure 5A, lane 2). A mixture of full-length and N-terminally cleaved L2 species was observed on some of the western blots. The cleaved species of L2 was found in foreskin- as well as cervix-derived particles from cultures immortalized by the 114/B and the 114/K HPV16 European isolates (Figure 5A,B). Primary foreskin rafts, which are HPV-negative, were used as a negative control (Figure 5A, lane 4 and Figure 5B, lane 5). Equal protein loading, as determined by Bradford assay, as well as overexposure of the blot did not yield any L2 bands in the negative control. We also analyzed virus particles derived from the previously characterized HPV16 cell line carrying a cysteine 428 to serine mutation in L1. The cysteine 428 residue plays a differentiation-dependent stabilizing role in mature HPV16 capsids [40,41]. These particles also had N-terminally cleaved L2 (Figure 5C, lane 2). This data suggests that the HPV16 L2 N-terminus is cleaved during virus assembly and maturation in tissue. The size of the cleaved L2 corresponds to the expected size from cleavage at the furin cleavage consensus site.

### 2.6. Infection of Primary Foreskin and Cervical Keratinocytes by HPV16 NV Derived from Either Tissue

**Figure 6:**
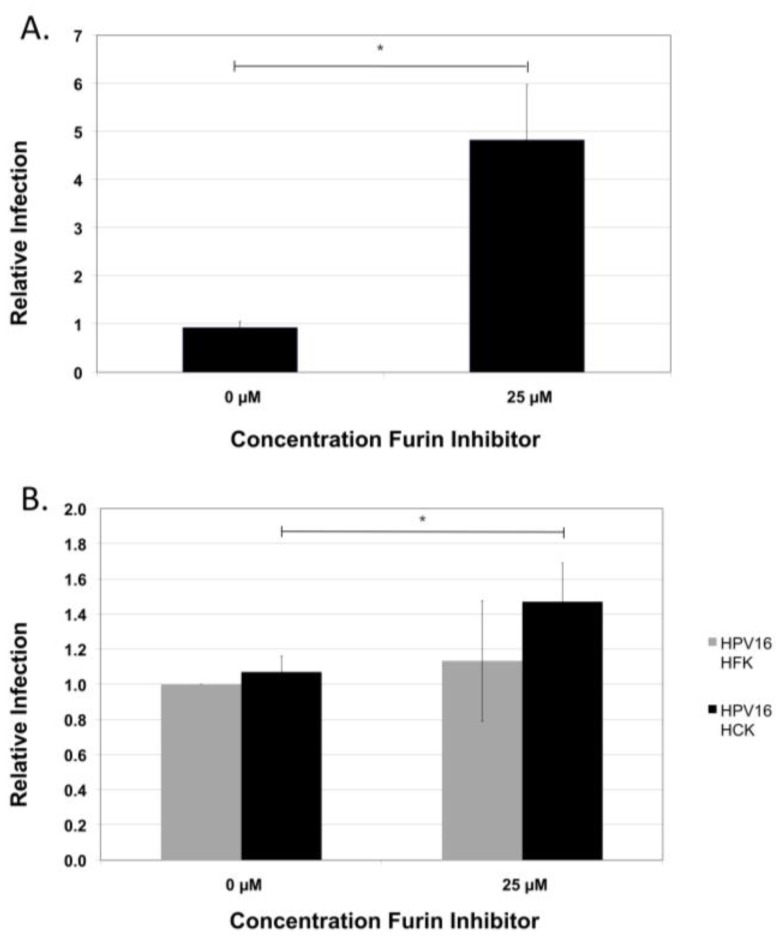
Furin-independent infection with cervical cell-derived HPV16. (**A**) Infection of HaCat cells with cervical cell-derived HPV16 in the presence or absence of 25 µM furin inhibitor; (**B**) Infection of primary keratinocytes with foreskin and cervical cell-derived HPV16 in the presence or absence of 25 µM furin inhibitor. Infections were analyzed by RT-qPCR measuring the relative amount of E1^E4 transcript two days post-infection normalizing to infections done in the absence of furin inhibitor. The results are expressed as the means of at least three independent infections utilizing at least two different virus preps and standard deviations are shown. Statistical significance (denoted by asterisks) was determined by student’s *t*-test and significance was defined as *p* ≤ 0.05.

To address the possibility that viruses produced in various tissues may mature differently and thus be exposed and processed differently by cellular PCs, we harvested HPV16 NV from stratified HPV16-immortalized cervical tissue (Figure 4G). Infections of HaCaT cells with HPV16 produced in HCKs were not blocked by the furin inhibitor (Figure 6A), similarly to the foreskin-derived virus (Figure 1A).

This supports the observation that HPV16 NV is independent of cellular furin and furin-related PCs for *de novo* infection and that it is not a cell line or cell-type specific effect. Next, we wanted to determine whether infection by HPV16 NV of primary keratinocytes is dependent on the activity of cellular furin. Infection of low-passage primary keratinocytes by HPV16 NV was not blocked in the presence of the furin inhibitor (Figure 6B), further confirming the observation that infection by HPV16 produced under physiologically relevant condition of stratifying and differentiating tissue is independent of cleavage by cellular furin for *de novo* infection. Taken together, these results demonstrate that HPV16 NV does not require cellular furin or furin-related PCs during infection of its host cells.

### 2.7. Conservation of the Furin Cleavage Site in L2

**Figure 7:**
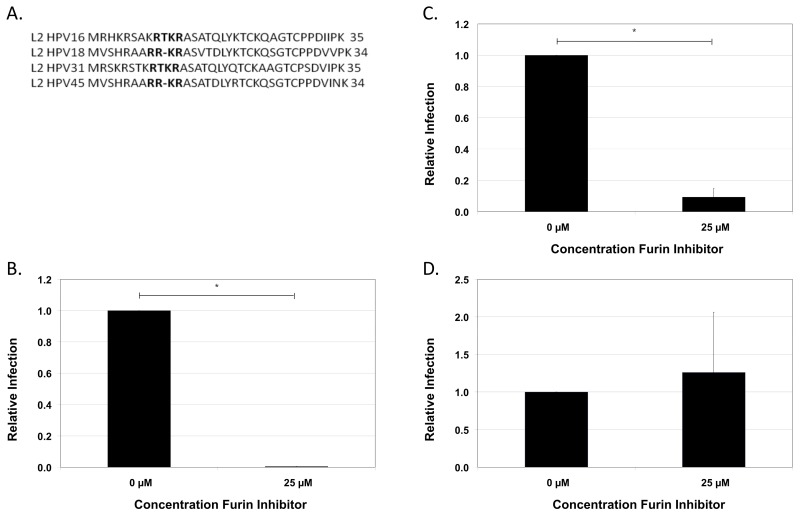
Furin inhibitory activities of other high-risk human papillomaviruses. (**A**) Alignment of the L2 N-terminal sequences of HPV16, HPV18, HPV31, and HPV45. A furin consensus site in each type is indicated in bold type. Infection of HaCaT cells with (**B**) HPV31 NV (**C**) HPV18 NV and (**D**) HPV45 NV in the presence or absence of 25 µM furin inhibitor. Infections were analyzed by RT-qPCR measuring the relative amount of E1^E4 transcript two days post-infection normalizing to infections done in the absence of furin inhibitor. The results are expressed as the means of at least three independent infections utilizing at least two different virus preps and standard deviations are shown. Statistical significance (denoted by asterisks) was determined by student’s *t*-test and significance was defined as *p* ≤ 0.05.

The consensus site for cleavage by cellular furin in the L2 N-terminus is highly conserved between the most common cervical cancer-causing HPVs; HPV16, HPV18, HPV31, and HPV45 (Figure 7A). We next sought to determine whether these high-risk HPV types necessitate cleavage by furin or a related PC for *de novo* infection of keratinocytes. The production of HPV18 and HPV45 virus from foreskin keratinocytes has been previously characterized [62,63]. Native HPV31 was produced from a cervical intraepithelial neoplasia type 1 biopsy-derived cell line CIN-612 9E [64]. Infection of HaCaT keratinocytes showed that HPV45 displayed a similar independence of cleavage by cellular PC as HPV16 (Figure 7D). In contrast, infections by HPV31 and HPV18 were clearly blocked in the presence of the furin inhibitor (Figure 7B,C).

## 3. Discussion

In this report, we demonstrate that tissue-derived genital high-risk HPV16 NV does not require the activity of cellular furin or related PCs during *de novo* infections. Importantly, a furin peptide inhibitor did not block infection by HPV16 and the virus could infect cells not expressing furin as efficient as cells that do express furin. Our finding was confirmed using virus particles harvested from foreskin- and cervix-derived organotypic cultures and by infections of cells harvested from tissues of both origins. This is important, as foreskin and cervix are both relevant tissues for the life cycle and transmission of HPV infections between males and females.

Furin is expressed at increased levels in the suprabasal cells of HPV-positive tissues, compared to expression of furin mainly in the basal layers of normal HPV-negative human foreskin and cervical tissues. This raises the possibility that the L2 protein of the virus may be cleaved by furin not just during *de novo* infections, but also during virion morphogenesis in the tissue. Furin, in its transmembrane form, is found trafficking in the trans-golgi network and a cleaved smaller version may be secreted. The accessibility of the furin catalytic domain may change as the cells are transiting through the epithelial cell layers and has been shown to be more readily detected by antibodies in the granular layer, where cells are progressing through terminal differentiation [56]. Here, the cells are going through remodeling and dissolution of membranous organelles and the Golgi apparatus [65,66,67]. Thus, reorganization of the normal inhabitants in the organelles would allow access to a different subset of substrates in the cells, including cytoplasmic substrates [56]. Virions are observed in the nuclei of differentiated cells as well as in the cytoplasm of de-nucleated cells [68], making it possible for assembled particles to be exposed to furin following nuclear degeneration. Indeed, the L2 N-terminus of immature HPV16 PsV can be cleaved prior to cell attachment *in vitro*. In contrast, the mature particles cannot be cleaved prior to cell attachment [25]. This suggests that the virions may go through a stage during maturation whereby the L2 N-terminus is exposed to cleavage by furin *in vivo* prior to the final maturation step. During tissue-differentiation, HPV16 NV goes through an extended period of increased maturation over a period of 20 days [40], during which the particles may be influenced by and interact directly with cellular factors [43]. Alternatively, the presence of the HPV viral genome in HPV16 NV may sufficiently alter the structure to allow for exposure of the L2 N-terminus during virion morphogenesis.

Cleavage of the L2 N-terminus is incomplete. *In vitro* furin cleaved PsV particles retain both full-length and to N-terminally cleaved L2 [50]. We also demonstrated incomplete cleavage of the L2 N-terminus in tissue-derived HPV16 particles. It is unclear whether the same particles contain the two types of L2 or if two distinct populations of particles exist. The physiological relevance of two forms of L2 being produced in differentiating tissue is supported by the demonstration of an L2 doublet by western blot from HPV11 particles derived from the athymic mouse xenograft system but not when the HPV11 L2 open reading frame was expressed alone. This was suggested to be a result of proteolytic cleavage of the full-length protein [61]. The increase in infection by HPV16 NV that is observed in the presence of the furin inhibitor suggests that there may be a threshold for the stoichiometric level of full-length versus cleaved L2 protein for efficient infection, and that additional cleavage during entry may have a negative impact on infection. L2 serves many functions in the HPV infectious process and the N-terminus of L2 is highly conserved between different HPV types. N-terminal cleaved L2 is responsible for the endosomal escape of L2 and the viral genome [25]. L2 then directs the retrograde trafficking of the viral genome to the nucleus [9,69]. Also important to note, is that the first 12 amino acids of the N-terminus of L2 are necessary for the interaction between L2 and DNA [70]. This supports a role for the cleaved as well as the un-cleaved form of L2 during HPV infection.

Cleavage of the L2 N-terminus occurs post-HS attachment on the host and *in vitro* furin cleaved HPV16 PsV is able to bypass the requirement of HS-binding to infect cells [50]. The GAG-independent infection by tissue-derived HPV16 has been previously reported [48,49]. This suggests a model where proteolytic cleavage of the HPV16 L2 protein during tissue differentiation allows the particles to bypass GAG-binding for primary attachment. This may permit the virus to bind directly to a functional entry receptor. However, it is also possible that various HPV types utilize diverse molecules for initial attachment to the host cell [49,71,72]. In addition, a requirement for GAGs during primary attachment is not predictive of furin-dependence for *de novo* infections. Tissue-derived HPV45 NV is dependent on the presence of cellular GAGs for primary attachment [48] but can infect cells *de novo* in a furin-independent manner.

We show here that HPV types 18 and 31 virions produced in differentiating tissue require the activity of cellular furin or a related PC during *de novo* infection, similarly to recombinant HPV PsV particles. Positive furin staining in the HPV18 positive tissue suggests that it is not the presence of furin in the tissue, but the exposure of the L2 N-terminus during virion morphogensis that is key. Unexpectedly, native HPV16 and HPV45 could infect cells independently of active cellular furin. These results warrant further studies focusing on the entry pathways of the individual virus types. The evolutionary related but genotypically and serologically distinct alpha HPVs may share many features of the virus life cycle but differ in some. It is interesting to note that different types of HPV PsVs have been reported to use distinct entry pathways under some experimental conditions but significant overlap in others [34,73,74,75,76,77,78]. Given the diversity of HPV types and their associated diseases [31], perhaps we should be more careful when simplifying and taking a broad view in regards to the general biology and infection path for distinct HPV types.

Infections with NV were performed with non-purified virus preparations. HPV particles are released with the de-nucleated cornified layer as the outermost cells are continuously shed into the surroundings [31,68]. Infections may take place with cell-associated virus or mechanically ruptured cells [68], which is likely to be the source of transmission to the genital epithelium during sexual activity. Thus, infections *in vivo* are unlikely to occur in isolation from cellular factors. A recent publication suggests that PsV particles may associate with cellular factors, including heparan sulfate proteoglycans and growth factors, to facilitate infection [79]. In addition, gradient-purification of virus particles may bias the analysis of a particular population of virus particles that may not represent the behavior of the population as a whole [40]. Further studies of purified NV particles would be warranted to analyze the biology of different subsets of virus particles to determine what cellular factors have a strong direct or indirect association with the virus particles and how that impacts the viral life cycle. The finding that HPV16 NV L2 N-terminus is proteolytically cleaved during virion assembly and maturation in a differentiation-dependent manner, supports the possibility that inhibitors of furin or related PCs may be effective to prevent the spread of genital HPV infections and is of great interest for future investigations.

## 4. Materials and Methods

### 4.1. Cell Culture and Virus Production

HaCaT cells were maintained in DMEM supplemented with 10% FBS and 1 mM sodium pyruvate. CHO FD11 cells and FD11 + furin cells were a gift from Stephen Leppla (National Institure of Allergy and Infectious Disease, NIH, Rockville, MD, USA) [55]. 293TT cells were maintained in DMEM supplemented with 10% FBS, 1 mM sodium pyruvate and 0.4 mg/mL hygromycin. CHO cells were maintained in minimal essential α-medium supplemented with 10% FBS and 200 µg/mL G418. Primary human foreskin keratinocytes from newborn foreskin circumcisions were isolated as previously described [62]. Primary human cervical keratinocytes obtained from cervical biopsies were isolated utilizing the same protocol. Primary keratinocytes were maintained in 154 medium (Cascade Biologics, Inc., Portland, OR, USA) supplemented with Human Keratinocyte Growth Supplement kit (Cascade Biologics, Inc., Portland, OR, USA). To create a HPV16-positive cervical cell line, the linear wild-type HPV16 (114/B) genome was electroporated into cervical cells as previously described [62] and stable cell lines were obtained. HPV18 [63] and HPV45 [62] stable cells lines were obtained in the same manner as previously described. The HPV31 (CIN-612 9E) cell line was obtained from a cervical biopsy [64]. Immortalized foreskin and cervical keratinocytes stably maintaining episomal HPV16, HPV18, HPV31, or HPV45 were maintained in monolayer cultures in E-medium in the presence of J2 3T3 feeder cells [64]. To produce native HPV virions, HPV-containing keratinocytes were grown in raft culture as previously described [64]. Mature virus particles were harvested from 20-day tissue by dounce homogenization in phosphate buffer (0.05 M sodium phosphate (pH 8.0), 2 mM MgCL_2_) [40]. All virus preps were treated with benzonase (375U) at 37 °C for one hour to remove un-encapsidated viral genomes [40]. Samples were adjusted to 1 M NaCl and centrifuged for 10 min at 10,500 rpm to precipitate cellular debris. HPV16 PsV produced using the HPV16 L1 and L2 codon modified p16LIw plasmid, packaging a GFP-expression plasmid (pfwB), was a kind gift from Patricia Day (Center for Cancer Research, NIH, Frederick, MD, USA) [80].

### 4.2. Virus Titers

HPV positive rafts were harvested and titers were determined using a qPCR-based DNA encapsidation assay against a standard curve using purified viral genomes for each HPV type as has been described [40,46]. Briefly, viral genomes were released by incubating 10 µL of each virus prep in a total of 200 µL HIRT buffer with 2 µL of 100 µg/µL proteinase K and 10 µL of 10% sodium dodecyl sulfate at 37 °C for 2 h. DNA was purified by phenol-chloroform-isoamyl alcohol extraction followed by ethanol precipitation and re-suspended in TE buffer. A SYBR green PCR kit (Bio-Rad, Hercules, CA, USA) and Bio-Rad CFX-96 Real-Time qPCR machine and software were utilized for PCR amplifications and subsequent data analysis.

### 4.3. Infectivity Assays

Furin inhibitor I (decanoyl-RVKR-chloromethylketone) was purchased from Calbiochem, Billerica, MA, USA. The HPV16 anti-L1 H16.V5 antibody was a gift from Neil D. Christensen. Neutralization and sensitivity of native virus to the inhibitor was tested using a previously described RT-qPCR-based infectivity assaying for levels of E1^E4 early viral transcript [40]. Briefly, cells were seeded in 24-well plates two days prior to infection. HaCaT cells were seeded 50,000 cells per well, CHO and 293TT cells were seeded 30,000 per well and healthy low-passage primary cells were seeded 70,000 per well to adjust for different growth rates. Antibody and/or inhibitor was mixed with virus and media one hour prior to addition to the cells and then incubated for 2 days at 37 °C. A multiplicity of infection of 10 was used for native virus infections unless otherwise noted. Total RNA was harvested two days post-infection with the RNeasy kit (Qiagen, Hilden, Germany). Primers and probes to amplify the E1^E4 viral target and TATA-binding endogenous cellular control target were previously described [40]. Amplifications were performed in duplicates for each sample 96-well qPCR plates (Bio-Rad) using the Quantitect probe RT-PCR kit (Qiagen) and the CFX-96 instrument (Bio-Rad). Relative levels of viral transcripts were determined by using the REST software. Results are representative of means and standard deviations of at least three independent infections for each virus type. Student’s *t*-test was performed with statistical significance calculated with *p* ≤ 0.05. Sensitivity of PsV to the furin inhibitor was tested by seeding cells and incubating with inhibitor the same way as described for native virus. Two days after the addition of PsV to the cells, PsV infection was assessed by immunofluorescent microscopy monitoring GFP-expression. Briefly, cells were washed extensively, fixed in 4% paraformaldehyde for 10 min at 4 °C, washed and mounted with and aqueous mounting media, Aqua Poly/Mount (Polysciences, Warrington, PA, USA). GFP expression was analyzed using a Nikon Eclipse 80i microscope. Images were captured using a Nikon Digital Sight SD-Fi1 camera using NIS-Elements 3.10 software (Nikon, Chiyoda, Tokyo, Japan).

### 4.4. In Vitro Furin Enzyme-Treatment of Virus Preps and Fluorogenic Furin Assay

Virus preparations were adjusted to 100 mM Hepes, and 1 mM CaCl_2,_ and 5U furin enzyme, followed by a 7 h incubation at 37 °C prior to infections [25]. As a control, cleavage of the Boc-RVRR-AMC fluorogenic peptide (Alexis biochemicals, San Diego, CA, USA) was assayed under the same conditions with the addition of 0.1 M 2-mercaptoethanol and 5% Triton X-100 at Ex/Em 350/450.

### 4.5. Histology and Furin Immunofluorescence Staining

Uninfected and HPV infected foreskin and cervical tissues were fixed in 10% neutral buffered formalin and embedded in paraffin. Sections (4 µm) were cut and either stained with hematoxylin and eosin (H&E) as previously described [64], or assessed for the expression of cellular furin. Briefly, tissue sections were de-paraffinized in xylenes 2 × 10 min. Slides were washed by 3 × 3 min in 100% ethanol to rinse xylenes, followed by rehydration in dH_2_O for 5 min. Antigens were retrieved by boiling in Tris-EDTA buffer (pH 9.0) for 10 min. Slides were allowed to cool to room temperature and rinsed in 0.05% TBS-Tween for 5 min. Samples were blocked with background sniper blocking agent (Biocare Medical, Concord, CA, USA) for 15 min at room temperature. Tissue sections were incubated with the furin primary antibody, MON-148, diluted 1:200 and/or the L2 antibody, RG-1, diluted 1:50 in DaVinci green antibody diluent (Biocare Medical) at 4 °C overnight. Slides were then rinsed in TBS-Tween 3 × 5 min before incubating with Alexa-Fluor 488 diluted 1:200 in DaVinci green antibody diluent for 1 h at room temperature. Slides were then stained with 10 μg/mL Hoechst 33342 (Molecular Probes/Invitrogen, Carlsbad, CA, USA) in TBS-Tween for 10 min at room temperature, followed by 2 × 5 min rinses in TBS-Tween. Slides were mounted with ProLong Gold Antifade (Invitrogen). Slides with secondary antibody only staining were used as controls for furin staining and HPV negative tissue sections stained with L2 as well as secondary antibody only stained slides were used as controls for L2 staining. Images of tissue sections were captured using a Photometrics CoolSnap cf2 camera and Nikon Digital Sight SD-Fi1 camera (Nikon, Chiyoda, Tokyo, Japan) using NIS-Elements 3.10 software on a Nikon Eclipse 80i microscope (Nikon, Chiyoda, Tokyo, Japan). Images of uninfected and HPV infected tissues were adjusted identically for brightness and contrast.

### 4.6. SDS-PAGE and L2 Western Blot

Equal aliquot (50 µL) from homogenized HPV16 virus preps and uninfected tissues were re-suspended in 6% 2-mercaptoethanol loading buffer and boiled for 10 min. HPV16 PsV was used for the detection of full-length L2 [50]. An L2 expression plasmid was used as a template to create a plasmid with an N-terminal deletion (amino acids 2–12), corresponding to the conserved furin cleavage site [25]. The p16L2h plasmid [81] was utilized as a template for site-directed mutagenesis using a QuikChange II XL site-directed mutagenesis kit (Stratagene, San Diego, CA, USA). The deletion was created using the follow complementing oligonucleotides: forward 5’ GTTATTACTTAACAATGGCATCGGCTACCCAAC 3’ and reverse 5’ GTTGGGTAGCCGATGCCATTGTTAAGTAATAAC 3’. The resulting plasmid was sequenced to verify the presence of the correct deletion. The HPV16 L2 N-terminal deletion expression plasmid was expressed by transfection in 293TT cells [82]. The resulting cell pellet was harvested by dounce homogenization on phosphate buffer similarly to the native virus harvest. Samples were loaded onto a 7.5% polyacrylamide gel followed by transfer onto a nitrocellulose membrane. Nitrocellulose membranes were blocked for 2 h at room temperature using Starting Block (Thermo Scientific, Waltham, MA, USA) with 0.05% Tween. To detect HPV16 L2, membranes were incubated overnight with the RG-1 monoclonal antibody (a kind gift from Richard Roden, Johns Hopkins, Baltimore, MD, USA) at a 1:200 dilution in blocking buffer. Membranes were then incubated with biotin-goat anti-mouse IgG (H + L) (Invitrogen) at a dilution of 1:10,000 for 1 h, followed by streptavidin horseradish peroxidase (HRP) conjugate (Invitrogen) at a dilution of 1:5000 for 20 min to amplify the signal. Membranes were washed extensively with PBS-T after each incubation step. HRP was detected using an ECL kit (Perkin Elmer, Waltham, MA, USA).
